# Staphylococci Culprits: Uncovering Microbial Contamination in Long-Term Care Shower Facilities

**DOI:** 10.1017/ash.2025.353

**Published:** 2025-09-24

**Authors:** Caitlin Crews-Stowe, Henry Spratt, David Levine, Abigail Nelson

**Affiliations:** 1 University of Tennessee at Chattanooga; 2 Dept. Biol., Geol., & Env. Sci., Univ. Tennessee at Chattanooga; 3 University of Tennessee/Erlanger Health System; 4 Deva Rea, AdventHealth

## Abstract

**Background:** Patient bathing plays a vital role in patient care and cleanliness, as well as in the prevention of infections, assisting in the removal of transient skin flora, which are predominately gram-positive organisms, particularly Staphylococci. However, there are inherent risks that come with the use of water in healthcare facilities, particularly the potential for acquisition of pathogenic bacteria from surfaces by residents. Current cleaning and disinfection protocols encourage the disinfection of the shower facilities in between each patient, but adherence to this practice can be a challenge with the staffing shortages seen in environmental services linked to long-term care facilities. The purpose of this point-prevalence study was to identify Staphylococci contamination in shower facilities at a long-term care and rehabilitation center **Methods:** Five shower room facilities in a long-term care and rehabilitation center were cultured on one day in July 2024. Five surfaces in the shower facility/room were cultured: the shower bench, the faucet, the floor drain, the grab bar, and the shower curtain. A total of 25 cultures were obtained using sterile transport swabs with liquid Stuart’s medium (Fisherbrand, Fisher Scientific, Suwanee, GA) and immediately placed in ice for transport to a microbiology lab. At the lab, the swabs were plated onto two different agars: CHROM MRSA agar and mannitol salt agar (MSA) and incubated for 48 hours. Plates were then read and assigned a qualitative “Yes/No” value if at least 1 colony forming unit of interest was seen. **Results:** Of the five surfaces cultured in the shower facilities, the bench shower seats were the most heavily contaminated. All of the bench shower seats tested positive for coagulase-negative Staphylococcus (CoNS) and Staphylococcus aureus (MSSA), and 40% of the bench seats tested positive for Methicillin-Resistant Staphylococcus aureus (MRSA). The floor drains were the second most contaminated surfaces, with 80% of surfaces testing positive for both CoNS and MRSA, respectively. Grab bars were the least contaminated surface examined, with only 40% of surfaces testing positive for MSSA and CoNS. **Conclusion:** This study showed that there was a high prevalence of Staphylococci organisms in the shower facilities, including showers that were marked “clean”. Finding 40% of shower benches contaminated with MRSA is concerning, particularly for some of the elderly patients using these showers. These findings highlight the lack of adherence to the cleaning and disinfection protocols. Further research should be done if adherence to their protocols will reduce Staphylococci surface burden.

